# The European Commission’s Green Deal is an opportunity to rethink harmful practices of research and innovation policy

**DOI:** 10.1007/s13280-022-01802-3

**Published:** 2022-11-02

**Authors:** Michael J. Bernstein, Thomas Franssen, Robert D. J. Smith, Mandy de Wilde

**Affiliations:** 1 Center for Innovation Systems & Policy, AIT Austrian Institute of Technology GmbH, Giefinggasse 4, 1210 Vienna, Austria; 2 Centre for Science and Technology Studies, Leiden University, P.O. Box 905, 2300 AX Leiden, The Netherlands; 3 Chisholm House, High School Yards, Edinburgh, EH1 1LZ UK; 4 University of Amsterdam, Postbus 15509, 1001 NA Amsterdam, The Netherlands

**Keywords:** Do no significant harm, Feminist science policy, European Green Deal, Research policy, Situated ethics, Sustainability

## Abstract

The European Union’s Green Deal and associated policies, aspiring to long-term environmental sustainability, now require economic activities to ‘do no significant harm’ to EU environmental objectives. The way the European Commission is enacting the do no significant harm principle relies on quantitative tools that try to identify harm and adjudicate its significance. A reliance on established technical approaches to assessing such questions ignores the high levels of imprecision, ambiguity, and uncertainty—levels often in flux—characterizing the social contexts in which harms emerge. Indeed, harm, and its significance, are relational, not absolute. A better approach would thus be to acknowledge the relational nature of harm and develop broad capabilities to engage and ‘stay with’ the harm. We use the case of European research and innovation activities to expose the relational nature of harm, and explore an alternative and potentially more productive approach that departs from attempts to unilaterally or uniformly claim to know or adjudicate what is or is not significantly harmful. In closing, we outline three ways research and innovation policy-makers might experiment with reconfiguring scientific and technological systems and practices to better address the significant harms borne by people, other-than-human beings, and ecosystems.

## Introducing ‘Do no significant harm’

Horizon Europe, the ninth research and innovation framework program of the European Commission, faces an uncomfortable reality: science and technology cause significant harm. This discomfort is driven by a much larger set of policies and measures advanced through the ambitious, urgently needed European Green Deal (European Commission 2019). At least 35% of the Horizon Europe budget, totaling some €35 billion, has been claimed as contributing toward the €1 trillion in funds to be mobilized to advance the Green Deal.^1^ Central to the instrument is a series of measures to harmonize private and public sector investment around a so-called ‘taxonomy’ of sustainable activities. Consequently, the 2020 Taxonomy Regulation directs European economic activities toward a set of environmental objectives and minimum social safeguards (European Commission 2020). The European Council and Parliament have affirmed six environmental objectives^2^: (i) climate change mitigation; (ii) climate change adaptation; (iii) the sustainable use and protection of water and marine resources; (iv) the transition to a circular economy; (v) pollution prevention and control; and (vi) the protection and restoration of biodiversity and ecosystem. On multiple occasions, the authors and expert groups informing adoption of the Green Deal’s Investment Plan and Taxonomy Regulation have acknowledged that scientific research and innovation fall within the remit of economic activities and thus will have to align with new environmental objectives. Hence the uncomfortable reality: scientific and technological practices and outcomes may not align with vital environmental objectives and, quite the opposite, may generate significant environmental harms.

According to the Taxonomy Regulation, qualification as ‘environmentally sustainable’ means that beneficial contributions of investments are not outweighed by harm to the stated environmental objectives. When it comes to Horizon Europe, as is often the case with Framework programs, the founding legislation espouses an ambition to substantially contribute to, “the creation and better diffusion of high-quality new knowledge, technologies and sustainable solutions…to address global challenges” (European Commission 2021b, p. L170/51). Some of Horizon Europe’s missions and clusters do seem well positioned to offer a ‘substantial contribution’ to the environmental goals presented above. But it is no longer sufficient to merely promise that public investments into research and innovation will bring positive outcomes; economic activities, including research and innovation, must now demonstrate they ‘do no significant harm’ to the six environmental goals set by the European Commission.

The Commission has published brief guidance on how to operationalize this new concept in research and innovation activities, but, as it stands, much remains unspecified. Reference to the ‘do no significant harm’ principle appears in Horizon Europe’s Pillar II clusters 4, 5, and 6 on digital, climate, and food research, respectively.^3^ Here the proposal template for the program, provided as guidance to researchers and innovators applying to Horizon Europe funding, asks that, where relevant, proposals show their “project will not carry out activities that make a significant harm to any of the six environmental objectives of the EU Taxonomy Regulation” (Standard Application Form (HE RIA, IA) part B, p.7, section on research methodology). Further, the principle will be considered in evaluation of proposals of the European Innovation Council program (European Commission 2021c), and possibly in Pillar II clusters in subsequent Horizon Europe biennial Work Programs. There is, however, a paucity of deeper support for researchers or innovators on how to enact this principle. Little more than a page of guidance is offered to researchers on precisely how to grapple with the question of (not) doing significant harm (European Commission 2021c).

In what follows, we consider some of the general tensions with the Commission’s current approach to operationalizing “do no significant harm” (DNSH) by looking at one of the few extant cases beyond R&I policy: nuclear power. After drawing out these illustrative tensions with the Commission’s current approach to DNSH in an established sphere of economic activity, we turn to the specific domain of research and innovation. We close by offering lessons from other attempts to govern science responsibly, suggesting these may be of use if R&I communities are to rise to the challenge of addressing the DNSH principle in their own policies, organizations, practices, and projects.

## Tensions in adjudicating harm^4^

To date, the Commission has prioritized tools such as environmental impact assessment and life cycle analysis to address the potential environmental harms of its Green Deal investments. The Parliament and Council have legislated that technical criteria to assess DNSH be based on available scientific evidence; comply at minimum with all EU environmental law; take account of life-cycle considerations; be updated regularly; include input from expert and relevant stakeholders; and defer when uncertain to the precautionary principle (in accordance with Article 191 of the Treaty on the Functioning of the European Union (European Union 2012)). Accordingly, technical expert groups charged with the development of the criteria for the DNSH principle have begun to articulate how we are to ‘know’ what constitutes harm. They have produced expansive guidance documents—running to 593 pages—that attempt to define possible areas of contribution and harm across numerous macroeconomic sectors (EU Technical Expert Group on Sustainable Finance 2020a, b). A technocratic and bureaucratic system, with its attendant politics, is thus in development to manage environmental harms as part and parcel of efforts to maintain control over environmental outcomes of EU economic activities.

While they draw on established approaches to policy making in the EU, these early attempts to quantify significant harm have sparked controversy. A prime example may be found in efforts to determine whether nuclear power should be included as an eligible activity under these new rules. The European Commission asked the Joint Research Centre (JRC) to provide a worked-through assessment of nuclear power considering the DNSH principle. The JRC deployed life cycle analysis and environmental impact assessment to determine whether using nuclear fission to generate energy meets the terms of the DNSH principle (JRC 2021). The JRC concluded nuclear fission complies with the requirement to substantially contribute, and do no significant harm, to the environmental objectives of the European Commission.

This conclusion was lauded by the Sustainable Nuclear Energy Technology Platform, a nonprofit promoting civil nuclear systems. Soon after, an expert response by three German federal organizations claimed that the JRC report adopted too-narrow a definition of environmental impacts. They argued that a, “scientifically comprehensive evaluation of using nuclear energy” (BASE 2021, p. 96) must also consider: the impact on future generations and participatory decision making in relation to disposal; the preservation of knowledge of radioactive waste repositories; proliferation issues; and uranium mining. This BASE report also critiqued the JRC approach for failing to acknowledge intergenerational concerns associated with the nuclear fuel cycle, concerns central to the sustainable development agenda foundational to EU climate policy and law (European Commission 2021d), as well as the participatory justice concerns of other aspects of EU environmental law, namely the Aarhus Convention of 2006 (Regulation (EC) No 1367/2006).

The nuclear power example highlights several tensions inherent to the technocratic approach taken by the European Commission to assess potential environmental harms. First, rather than developing new methods to enact new policy concepts, ‘Do No Significant Harm’ is being operationalized using established and technical approaches to assessing what counts as harm. Thus, what was originally articulated in quite profound terms, akin to the Hippocratic oath or the precautionary principle, in practice is relying on dominant modes of thinking and doing (Dunlop 2010; Stirling 2016). Given that such dominant modes of thinking and doing bear responsibility for generating significant harms in the first place, turning to them to also determine what counts as harm is likely to prove insufficient.

Second, attempting objective, definitive quantitative assessment of something as contextual and relationally mediated as harm—let alone its degree of significance—exerts a modernist logic of control on phenomena that exceeds the boundaries of scientistic and technocratic ways of knowing. Matters of harm and their significance are questions that emerge at the intersections of social, technical, political, and environmental concerns of a multitude of actors. As such, matters of significant harm invite high levels of imprecision and ambiguity or uncertainty; states that will all change over time (Jasanoff 2003; Sarewitz 2016). What kind of harm is relevant? On which timescales? For whom do the countervailing benefits accrue? And who gets to decide what the thresholds of significance are? Such questions—embedded as assumptions in determining the system boundaries of any life cycle analysis (Wender et al. 2014)—are contested. Questions of significant harm are therefore always ‘trans-scientific’ (Weinberg 1972); ones that, despite appeals to modernist approaches, will resist reduction, require situated interpretation, and demand debate.

The question of appraising environmental harms from technologies associated with nuclear power cannot be reduced to a simple binary of such technologies either causing or not causing harm. Similarly, assessments of harm cannot be understood as solely technical procedures, however, robust employed assessment methods may be. While life cycle analysis and impact assessment are often understood to be able to produce ‘objective’ knowledge (Daston and Galison 2007), they can be better understood as tools to build up partial pictures of inherently uncertain and ambiguous contexts (Haraway 1988). Employed judiciously, they can illuminate assumptions and value judgements to inform decision-making (Boucher et al., 2014; Raman et al. 2015). But when used as a form of regulatory science to adjudicate what is or is not ‘significantly harmful’, they are likely to prove woefully inadequate, sparking contestation and potentially undermining the legitimacy of the governmental institutions purporting to care about said harms (Jasanoff 1990; Wynne 1991). The failed technocratic attempts of the U.S. Department of Energy, for some 50 years, to permanently store high-level commercial nuclear waste offer a case-in point. Taking consideration of nuclear waste alone, questions of siting permanent high-level waste storage facilities in democracies involve issues of equity (of processes and outcomes); trust; community well-being, information, and economic, needs; political power in negotiations; and flexibility and adaptability to dynamic societal needs, circumstances, and values over extended periods of time (Richter et al. 2022).

In adopting an eco-modernist perspective in which environmental harms are understood as outcomes of a market economy to be solved by governmental policy (Andersen and Massa 2000), the DNSH principle as enacted by the Commission to date advances strong claims on the knowability of harm, its boundaries, its impacts, and its manageability. This is well highlighted by the BASE critique of the JRC report regarding, for example, the choice to ignore psycho-social secondary illnesses, and loss in quality of life, social cohesion, and economic prosperity associated with nuclear accidents when issuing its finding on significant harm—all observable impacts, even if not quantitatively measurable through direct radiological effects. As enacted, the DNSH principle privileges technocratic actors to name and judge the significance of harms. Further, it equates and normalizes ideas of benefit and harm across vastly different environmental objectives and contexts.

Given the challenges surfaced by the first major technocratic operationalization of DNSH in a contested but well-established technological system like nuclear power, how might one think with the principle for much more uncertain, ambiguous, and arguably unknowable situations as those futures brought about through public investment into research and innovation? As we explain below, an alternative and potentially more productive approach would be to move away from attempts to unilaterally or uniformly claim to know or adjudicate what is or is not harmful. We suggest instead to treat the ambition to do no significant harm as an invitation to reflect, debate, and respond to how harm might unfold in specific, situated contexts. This approach draws on analytic traditions developed in (feminist) science and technology studies, explored below.

## An alternative approach to understanding harm

Feminist studies of science and technology show that research is always situated in particular concerns (Haraway 1988; Harding 1991). The notion of *situatedness* is an argument for positioning, for grounding knowledge ‘somewhere,’ whether in our research practices as scientists and scholars or our research evaluation practices as policy practitioners. Situatedness operates by foregoing the ideal of a neutral viewpoint from which one can determine, once and for all, whether research is beneficial or harmful, and instead foregrounds, “negotiating specific, situated concerns” (Mol and Hardon 2021, p. 187). Such negotiations necessarily depend on who asks what questions, who is invited to answer, and in what manner. Because they emerge from particular situations, harms cannot be known up-front. Situating, therefore, entails being able to ‘stay with’ the difficult issues foregrounded by addressing questions of significant harm or substantial benefit of science and technology development, as well as attending to the voices that are excluded from negotiations (Giraud 2019; Haraway 2007).

Seen in this light, DNSH invites a situated ethics^5^ approach in which past, present, and future environmental harms (and social exclusions) may be tended in ways that allow diverse groups of stakeholders to stay with the challenge of “crafting more complex notions of ethical responsibility” (Giraud and Hollin 2016, p. 45; see also Puig de la Bellacasa 2010, 2011). Situating significant harm would invite recognition that environmental harms perpetrated in the past persist *to this day*, and that forecasted planetary harms *are already* perpetrated on many populations and ecosystems (Whyte 2016). Such inter-temporal harms exist as a form of violence borne not only by the land, but also by human and nonhuman bodies, in ways often amounting to state-sanctioned suffering (Murphy 2020).

The European research and innovation system is situated amidst vast networks of relationships (Puig de la Bellacasa 2011). For instance, globalized supply chains and resource circulation makes it far easier than ever to displace harm geographically, amidst a long history of human-caused environmental degradation (Jarrige and Roux 2020). As a stark example, one might observe harm done by shipping electronic waste from U.S. and Europe to Giyu, China, displacing toxic burdens onto young children, pregnant women (Chen et al. 2011). A situated approach to harm also foregrounds the production of harms over these extended geographies and scales. By tracing supply chains, systems of extraction and networks of production in detail, it becomes possible to see and build—with funding mechanisms for instance—alternative, potentially less harmful, modes of research (Herrero et al. 2015).

In addition to being better suited to the extended temporalities of harm, situating in physical (and virtual) places offers an opportunity to redress harms in human and more-than-human *relationships*, and also better approach questions of “significance.” Devoting resources to figuring out how to transfer burdens of harm onto perpetrating relations, rather than the ecosystem and human and other-than-human bodies, represents a novel course of action afforded by a situated approach to significant harm in European research and innovation policy. If such relations recognized the situatedness of people and places beyond factors like potential employment or economic return, would determinations of “significance” be so quick to legitimate and displace potentially devastating environmental or social harms? Acknowledging human and ecosystem interdependencies through a situated approach (Murphy 2020), or adopting other forms of knowing and relating (e.g., Salmon 2000)—while still valuing potential positive contributions of scientific and technological development—might support vital steps towards reconciling past and mitigating future harms.

## Situating significant harm in research policy

Using a situated approach requires a reformulation of how to appraise significant harm, namely as concerns negotiated from specific temporal, locational, and species standpoints. A situated approach is not just a theoretical proposition; it affords practical actions research and innovation policy makers or program managers might pursue. Below, we offer three foundations on which to build a situated approach to navigating questions of “significant harm” in research policy and practice.

## Diversifying significant harm

Early indications from the European Commission (e.g., European Commission 2021c, e) are that individual applicants to funding programs will be tasked with explaining how their proposed projects comply with the DNSH principle. Individuals and small groups of technical experts are thus likely to be the ones deciding what is or is not harmful to whom and in what context. As we have seen in the nuclear case, this approach is likely to produce a narrow and democratically illegitimate understanding of the appropriate concerns associated with a scientific or technological trajectory that will be open to contestation and controversy.

A situated approach to DNSH would start by taking a broad gaze with regards to determining relevant temporalities, geographies, and species potentially harmed, and take-seriously the need for a plurality of perspectives when making these judgements. As such, adopting a situated approach entails including in these appraisals people with diverse knowledges and experiences who claim relation to a particular research arena. This approach would deprivilege scientific and technical voices in saying what might be harmful, how, for whom; enable understandings of harms enriched by the experiences of those actually affected; and produce more democratically legitimate appraisals in systems that claim to work in service of public value (Bozeman and Sarewitz 2011).

This recommendation builds on widespread attempts in research and innovation governance to integrate more diverse knowledge and experiences into the structures of research policy, processes, and practice (e.g., open innovation, open science, citizen science, multi-actor engagement, human-centered design, responsible innovation, etc.). In adopting a situated approach to DNSH, such impulses could be strengthened by extending to relations—species and ecosystems—burdened by prior and implicated by future harms (Szymanski et al. 2021). Attend seriously to equalizing differences in political and economic power such that new voices be not only included but also heeded.

## Allowing for ambiguity

The presence of the word ‘significant’ in ‘do no significant harm’ implies a degree of ambiguity. However, as we have outlined above, the tendency in European research policy seems either to adjudicate against ‘significantly harmful’ technological trajectories or, more commonly, to delegate the task of appraising harm onto funding applicants as a form of technocratic compliance. There may be situations in which harms are unanimously agreed upon and can thus be adequately translated into compliance mechanisms—the use of particular ‘forever chemicals’ or particular biological feedstocks in research for instance. However, the majority of harms are likely to be ambiguous, existing to degrees. Rather than ‘removing’ ambiguity through calls for compliance, a situated approach to operationalizing DNSH in research policy would encourage engagement with the ambiguity of harm.

Here our recommendation is to expand peoples’ capabilities to meaningfully engage with situated aspects of significant harm. Standard funding organization initiatives in capacity building offer a useful starting point—networking series, seminar series, webinars, trainings, etc. These could be augmented with efforts to socialize policy practitioners and researchers with other relations (people, ecosystems and species burdened by harms) to reflect on notions of harm and significance across time and space, outside of the proscribed settings of scientific projects and agendas. Building capacity outside of projects could be complemented by resourcing social scientific or humanities scholars not only to join projects grappling with questions of significant harm but also to apply qualitative methods to enrich the knowledge base around ways for funding and research performing organizations to situate significant harm. The benefits of such approaches to encouraging engagement with the ambiguity of harm would likely be many and varied: from ensuring the existence of a community able to respond to requirements to consider DNSH, to identifying potential organizational blockages and the options for re-design to meaningfully stay with harms as they emerge and change over time.

## ‘Staying with’ significant harm

A situated approach to engaging with DNSH in research policy would focus on the relations from which harm emerges, taking as given that, relationally, some harm is *inevitable*. The salient questions here shift from ones of identifying and avoiding harm, to understanding and making visible what harms are done and to whom, while focusing on developing positive, constructive relations in spite of these harms (Szymanski et al. 2021). Additionally, in the context of science and technology programs, harms and their distribution are likely to change over time. As research develops, new harms may become visible. Incoming staff will bring new competencies to appraise and engage with harms. Significantly, decisions can be made over the course of scientific research and technology development to ameliorate previously identified harms. A situated approach to DNSH would therefore prioritize methods that enable researchers and administrators to ‘stay with’—that is to keep sight of and continuously engage—the harms related to their work over time.

In practice, staying with harms requires unambiguous resource commitments on the part of funders, but it can also be incentivized through evaluative processes such as grant review and monitoring. Policies that legitimate the labor of engaging with these harms over time would be needed. This labor may be emotional—in the case of animal work, for instance (Giraud and Hollin 2016). Or it may be scientific in the sense of requiring new research practices, analyses, or infrastructures—as has been the case with fields of research such as toxicogenomics (Fortun 2005) and ecotoxicology (Roberts et al. 2008). In addition, ‘staying with’ means removing pressures to turn away from questions of significant harm. This might mean reconsidering the perverse effects of quantitative metrics common in research governance: quests for more publications, patents, more people engaged, dissemination and communication statistics, academic citations, re-tweets, and the like are going to be of little help (Benessia and Funtowicz 2015; Wilsdon et al. 2015). Such pressures mean that community partners (often the ones harmed, to say nothing of species and ecosystems) rarely get what they might need from a project. The undercurrent of anxiety of not being perceived, according to contrived quantitative metrics of ‘excellence’ distracts and blocks outright researchers and policy practitioners from valuing the likely slow and patient work of staying with questions of significant harm, and (re)discovering ourselves as people situated in webs of relations to other people, species, and ecosystems (see Franssen 2022).

## Conclusion

Giving prescriptions for “dealing with” significant harm—as if it were invariant and absolute—runs contrary to the premise of a situated approach (although we are sympathetic to the impulse). Nevertheless, our general invitations for how to enact a situated approach can be pragmatically pursued in a number of ways and for a range of benefits—with the caveat that these invitations will need tailoring to actions and situations. We summarize the above invitations for a situated approach to the “do no significant harm” principle in R&I activities in Table 1, below. The likely targets of these pursuits include, but are not limited to, administrative practices of research policy: advisory boards, research agendas, proposal guidance, proposal evaluation, professional development opportunities as well as monitoring and evaluation and intellectual property rights regimes. Should the EC invest in studying and experimenting with alternative approaches to implementing the DNSH principle, a broad and lengthy empirical program can easily be imagined, from which further guidance could be developed.

**Table 1:** Summary invitations for a situated approach to pursuing the ‘do no significant harm’ principle

Invitation	Pragmatic pursuits	Benefits on offer
Diversifying significant harm	Expand timespans, spaces, species, and human perspectives considered whenever identifying and characterizing harms Actively listen to the new perspectives and voices included	Enriched understanding of harm by including those affected More legitimate appraisals of harm Fairer processes and outcomes (e.g., reduced political and economic power asymmetries)
Allowing for ambiguity	Socialize researchers, research funding organizations, stakeholders and other science and innovation actors on the ways harm is ambiguous, dynamic, yet still addressable Resource people and their organizations to identify barriers and innovate to address significant harms as they emerge and change over time	Enhanced research and innovation (R&I) community capacity (i.e., human resource) Higher quality implementation of DNSH in R&I programming Greater likelihood of creative, meaningful approaches to address significant harms
“Staying with” significant harm	Actively reduce pressures to ignore “significant harms” that may seem to “get in the way” of program, project, organization, or career goals Support slow, patient approaches to building cross-disciplinary and more-than-human relationships (i.e., not only with a range of stakeholders, but also species and ecosystems)	Freedom from quantitative performance anxiety Reduced conflicts of interest where people “stay the course” and ignore, rather than “stay with” the harm Greater likelihood of cultivating adaptive approaches to ambiguous and dynamic situations where significant harms emerge

If anything, the above invitations are modest in their suggested reconfigurations. Experimentation is the core of scientific approaches to knowledge development. Our proposal invites funders, scientist, social scientists, and others to extend this commitment to experimentation to our practices of governing research and innovation (e.g., see Smith et al. 2021). We invite researchers, innovators, stakeholders, and policy makers to wonder: what might happen to our innovation governance regimes were we to take seriously the challenge of situating significant harm? We invite them to approach this question not with the same eco-modernist techniques that perpetuate harms, but rather with attempts at something different. What happens to our research and innovation systems when relations among humans, species, and ecosystems are held in much higher regard than the contemporary paradigms of extraction, substitution, or disposability? The above invitations are not about abandoning scientific and technological pursuits; rather they are about taking the opportunity to put researchers’ ingenuity and spirit to the cause of reforming scientific and technological cultures toward ones that tend, rather than unthinkingly (or resignedly) perpetuate, significant harm.

We offer these proposals as an invitation to experiment radically, in a manner commensurate to the urgency borne of crises social, ecological, and climatic—crises shaped by the scientific and technological projects of research and innovation themselves (c.f., Kates 2001; Rockström et al 2009; Westley et al 2011). The outcomes of such efforts need not be known in advance, hence our invitation to experiment. A funding organization might start by sandboxing portions of its portfolio to critically reflect on questions of harm *before* even setting an agenda; might open processes to representatives of future generations, impacted ecosystems and species; might commit in advance to funding such experiments for a number of years, resourcing not only the work but also the capacity building and thoughtful monitoring to see how notions of harm begin and dynamically change over time and space. These must include a commitment to learning from failures without retreating to the familiar modes of technocratic assessment that reproduce the very harms the DNSH principle affords a chance to rectify.

Facing questions of significant harm with due gravity is vital because another way of looking at research and innovation—and economic activity more broadly—is the following: a history of investments that, essentially, hand out rights to harm people and ecosystems in the name of promises for what is just over the horizon of discovery (or profit). While the Commission has notably strived to invite more open and inclusive ways of doing research, for example through responsible research and innovation, efforts to institutionalize these policies are short-lived, do not focus on broader economic activity, do not address environmental harms, and do not approach the challenge of addressing scientific and technological issues through a situated, relational lens capable of modulating research cultures over time (Novitzky et al. 2020). While environmental laws abound in Europe, these, too, are often insufficiently funded, monitored, and enforced; do not privilege relations with other-than-human beings or ecosystems when considering harms; and are framed in opposition to, rather than within the heart of, economic activities (Darpö 2021). Hence the great promise of the European Green Deal and the ‘do no significant harm’ principle—being situated at the heart of requirements for economic activity. Hence the great peril should it be applied technocratically, granting greater permission for environmental degradation rather than inviting critical reorientation of the relationships in which people, species, and ecosystems must coexist.

Taken seriously, a situated approach would mean *earning* the legitimacy to make claims of harm or significance. It would also mean *earning* the right for promises of benefit to be legitimate. As Fortun wrote, referencing the case of genomics, “Having a right to make promises will have entailed learning to live with, and cultivate, the excesses of promising” (2005, p. 171). To acknowledge the broad sweep of environmental and human harm committed by peoples and governments currently under the banner of the European Union would be to reckon with the fact the Commission may not yet have earned the right to make decisions about the significance of harm in the face of scientific and technological promise.^6^ A situated approach acknowledging this past—instead of marshalling culpable scientific and technological methods to technocratically re-validate and re-perpetrate harm—would represent a vital step toward earning this right.

## Footnotes

1 Per the Sustainable Europe Investment Plan, European Commission (2021a, p. 7).

2 Social objectives are not elaborated in the regulation. Instead, the text references need for observing minimum safeguards related to human and labor rights, as found in the Charter of Fundamental Rights of the European Union, the OECD Guidelines for Multinational Enterprises Principles on Business and Human Rights; and International Labor Organization Fundamental Principles and Rights at Work.

3 Making up a combined 35% of the Horizon Europe budget.

4 Throughout the text, when we use the word “harm” instead of the legal term “significant harm”, we imply “significant”. The issues raised in the text apply equally to “significant harm” and to questions of adjudicating the difference between either.

5 While there are discussions within feminist technoscience on what exactly constitutes a situated ethics (c.f., Martin et al. (2015)), various authors agree on how a situated approach to knowledge production foregrounds relationality. This differs from more modernist—and predominant—approaches to ethics, which foreground universality and objectivity of ethical principles (see Latour 1993; Puig de la Bellacasa 2010, p. 162; Mol and Hardon 2021, p. 186). Situatedness, we go on to argue, is essential to meaningfully determining significant harms in the specific contexts in which they are perpetrated or emerge.

6 Take, for instance, the loopholes carved into other environmental regulations of the Commission, in which research and development activities are granted whole-scale exemption from responsibility for things like pollution prevention (c.f., (Directive 2010/75/EU)).
